# N100 Repetition Suppression Indexes Neuroplastic Defects in Clinical High Risk and Psychotic Youth

**DOI:** 10.1155/2016/4209831

**Published:** 2016-01-12

**Authors:** Joseph Gonzalez-Heydrich, Michelle Bosquet Enlow, Eugene D'Angelo, Larry J. Seidman, Sarah Gumlak, April Kim, Kristen A. Woodberry, Ashley Rober, Sahil Tembulkar, Kyle O'Donnell, Hesham M. Hamoda, Kara Kimball, Alexander Rotenberg, Lindsay M. Oberman, Alvaro Pascual-Leone, Matcheri S. Keshavan, Frank H. Duffy

**Affiliations:** ^1^Boston Children's Hospital, 300 Longwood Avenue, Boston, MA 02115, USA; ^2^Department of Psychiatry, Harvard Medical School, 401 Park Drive, Boston, MA 02215, USA; ^3^Department of Psychiatry, Commonwealth Research Center, Beth Israel Deaconess Medical Center, 75 Fenwood Road, Boston, MA 02115, USA; ^4^Department of Psychiatry, Massachusetts General Hospital, 55 Fruit Street, Boston, MA 02114, USA; ^5^Department of Neurology, Harvard Medical School, 25 Shattuck Street, Boston, MA 02115, USA; ^6^Neuroplasticity and Autism Spectrum Disorder Program and Department of Psychiatry and Human Behavior, E.P. Bradley Hospital and Warren Alpert Medical School, Brown University, 1011 Veterans Memorial Parkway, East Providence, RI 02915, USA

## Abstract

Highly penetrant mutations leading to schizophrenia are enriched for genes coding for N-methyl-D-aspartate receptor signaling complex (NMDAR-SC), implicating plasticity defects in the disease's pathogenesis. The importance of plasticity in neurodevelopment implies a role for therapies that target these mechanisms in early life to prevent schizophrenia. Testing such therapies requires noninvasive methods that can assess engagement of target mechanisms. The auditory N100 is an obligatory cortical response whose amplitude decreases with tone repetition. This adaptation may index the health of plasticity mechanisms required for normal development. We exposed participants aged 5 to 17 years with psychosis (*n* = 22), at clinical high risk (CHR) for psychosis (*n* = 29), and healthy controls (*n* = 17) to an auditory tone repeated 450 times and measured N100 adaptation (mean amplitude during first 150 tones − mean amplitude during last 150 tones). N100 adaptation was reduced in CHR and psychosis, particularly among participants <13 years old. Initial N100 blunting partially accounted for differences. Decreased change in the N100 amplitude with tone repetition may be a useful marker of defects in neuroplastic mechanisms measurable early in life.

## 1. Introduction

Schizophrenia (SZ) is a progressive disorder, with the prodromal or clinical high risk (CHR) phase evolving into full psychosis within the first three years of ascertainment in approximately one-third of affected individuals and remitting or remaining stably symptomatic in the remaining two-thirds [1, 2]. Although only a minority of patients in the CHR phase develop SZ, most have marked limitations in mental, cognitive, and emotional functioning that lead to clinical referral. Moreover, these deficits often accrue over time, regardless of ultimate diagnosis, resulting in significant functional impairment [3]. Discovering biomarkers sensitive to the prodromal phase may improve treatment by assisting in the identification of at-risk individuals so that interventions may be applied early, thereby delaying or preventing the emergence of psychosis and minimizing functional impairments, including among those who do not progress to a psychotic disorder [4, 5].

Accumulating evidence suggests that defects in the molecular pathways subserving synaptic plasticity, including long-term potentiation (LTP) and long-term depression (LTD) processes, play an etiological role in SZ and thus, by extension, in the CHR phase [6]. Postmortem samples from patients with SZ show low spine densities on the basilar dendrites of pyramidal neurons in various cortical regions and altered levels of mRNA for proteins in the LTP pathways in dendritic boutons, including genes in the CDC42 signaling pathways and Neuregulin 1 and its receptor [7, 8]. Additionally, large genomic studies of both common and rare mutations associated with SZ have independently implicated glutamatergic neurotransmission and synaptic plasticity [9]. These genes include components of the N-methyl-D-aspartate receptor signaling complex (NMDAR-SC) as well as genes with effects on plasticity presynaptically, thus pointing to a broad association of risk with synaptic regulation [10].

Defects in brain plasticity mechanisms could impact neurodevelopment via synaptic pruning processes. Current views on the mechanisms by which synapses are eliminated during brain development hypothesize that malfunction in pathways leading from NMDA-type glutamate receptors could lead to excessive synaptic pruning in adolescence [11–14]. Notably, neuroimaging studies of adolescent and young adult patients with first-episode SZ have implicated enhanced synaptic pruning in the development of psychosis [15, 16].

Before much of these genetic and neuroimaging data were available, acute administration of NMDAR antagonists was observed to induce symptoms that closely resemble those of SZ, including negative symptoms [17]. This observation led to the testing in preclinical models of compounds that enhance NMDAR activity, including metabotropic glutamate receptor agonists, glycine receptor agonists, and glycine reuptake inhibitors. Most studies of these compounds were performed in patients already afflicted with SZ with the aim to increase NMDAR dependent activity and, consequently, decrease SZ symptoms, especially negative symptoms, which are not alleviated by conventional antipsychotics. After demonstrating some promise in pilot studies, these agents failed in subsequent large placebo controlled trials [18]. Because earlier stages of SZ might be more amenable to treatment, attempts to use NMDAR modulators in the CHR period have also been undertaken, with some success in pilot studies. For example, D-serine treatment has been shown to decrease negative symptoms in CHR patients [19]. Larger, placebo controlled trials are needed to test these agents in CHR patients, as establishing the efficacy of therapies meant to enhance NMDAR functioning has proven more difficult than expected.

Noninvasive measures of neural plasticity could facilitate this process in several ways. For example, plasticity measures could signal if patients to be included in clinical trials of these agents have measurable deficits in neural plasticity. These measures could also signal if experimental therapies are improving neural plasticity as predicted by preclinical studies. Additionally, since plasticity pathways are active early in brain development, measures of their dysfunction could identify individuals in early life who are at particularly high risk for developing CHR or PS (e.g., among patients at high genetic risk). Finally, these measures could be utilized to evaluate the ability of experimental treatments to reverse plasticity deficits that emerge prior to the onset of CHR or PS symptoms [20].

Methods for measuring neural plasticity are in development, and several have been used to demonstrate neural plasticity deficits in patients with CHR or SZ [21]. Such methods include measuring changes in cortical response after transcranial magnetic stimulation (TMS) [22, 23] and in cortical evoked response potentials (ERPs) after high frequency repetitive sensory stimulation [24, 25]. Notably, ERP-based measures of automatic memory formation and deviance detection have been studied extensively, are plasticity dependent, and are abnormal in CHR and SZ. Moreover, they have shown promise as predictive biomarkers for transition from CHR to psychosis. Specific ERP waveforms that have demonstrated utility in this context include the P300 [26] and the mismatch negativity (MMN) [27]. Both of these responses are elicited by exposing the participant to a repetitive standard sensory stimulus and to a randomly interspersed rare stimulus (the “oddball” stimulus) that violates the regularity of the standard stimulus. To elicit the P300, the participant is asked to attend to the stimulus; no such instruction is given when eliciting the MMN [28]. While its application to pediatric age patients with CHR or SZ has been limited, MMN has advantages over the P300 as a translational measure of plasticity. It does not require participant cooperation, has been studied in newborns, children, and adults, and has an analog measureable in rodents [29, 30]. However, the MMN is hypothesized to sum two different plasticity mechanisms, that is, sensory specific adaptation and deviance detection. Furthermore, in rodent models, NMDAR antagonists differentially affect the amplitude of the response to the standard stimulus and to deviance detection, depending on the dose of NMDA antagonist administered [30]. These findings argue for the potential utility of measuring sensory specific adaptation separately from deviance detection.

Another ERP measure of cortical auditory response, the N100, shows promise as a biomarker of neural plasticity that can be measured very early in development. The N100 response is generated in the auditory cortex approximately 100 milliseconds after an auditory signal. It occurs prior to the MMN and represents an earlier and simpler aspect of sensory processing. Moreover, the amplitude of the N100 decreases with repetition of a tone [28]. This decrease reflects sensory specific adaptation that is not confounded by deviance detection and thus provides a purer measure of plasticity mechanisms subserving this phenomenon than MMN [30]. Furthermore, numerous studies have established that the N100 amplitude is decreased in SZ [31–37]. Deficits in the N100 and in its adaptation with repetitive presentation of an auditory stimulus in CHR and SZ have also been described previously, though to our knowledge not in exclusively pediatric age samples [4, 28, 34, 38]. Importantly, the auditory N100 can be measured beginning in early childhood [39] and thus may be useful in therapeutic trials aiming to reverse processes leading to SZ very early in development. Moreover, the N100 is measurable in rodents and thus could provide a translational bridge between preclinical studies in rodents and human studies of potential therapeutics [40–42].

The mechanisms of N100 repetition suppression are only partially understood. Several prefrontal, cingulate, and parietal lobe regions exhibit stronger N100 repetition suppression than the auditory cortex, implying that neural networks underlying repetition suppression include these regions and that the initial N100 response to stimulus and its suppression may involve separate mechanisms [43]. Notably, N100 repetition suppression is dependent on baseline N100 amplitude [31–33, 36]. Administration of the NMDR antagonist phencyclidine (PCP) induces SZ-like deficits in the N100 amplitude and dependence on stimulus repetition rate that parallel those observed in SZ [28, 44]. Thus, the deficits in sensory adaptation in SZ and CHR indexed by N100 repetition suppression may be due to weaknesses of synaptic plasticity mechanisms in frontal, cingulate, and/or parietal-temporal connections, low baseline N100 amplitude, or both.

Together, these data suggest that N100 repetition suppression may be a useful biomarker of target engagement by experimental therapies aimed at improving NMDAR functioning [45]. Determining whether N100 repetition suppression is altered in CHR and PS present in early life is critical for evaluating the measure's usefulness as a clinical tool. The goal of the current study was to test whether N100 repetition suppression shows a gradient of deficit that parallels the gradient of clinical severity of psychotic symptoms by comparing healthy control (HC) participants to patients with CHR or PS in a fully pediatric sample. Additionally, clinical group differences in the N100 repetition suppression response were considered separately in subsamples of participants between 5 and 12 years and 13 and 17 years of age to explore whether very early presentation of CHR or PS is accompanied by more marked plasticity deficits. Research suggests that very early onset psychosis (i.e., emerging before the age of 13 years) shows more severe premorbid neurodevelopmental abnormalities and poorer treatment response and outcomes than later onset psychosis [46–48]. We hypothesized a progression in plasticity dysfunction from HC to CHR to PS groups. We further hypothesized that the plasticity dysfunction would be more robust in the CHR and PS groups within the younger subgroup than in the older subgroup of participants.

## 2. Methods

### 2.1. Participants

**Figure 1:**
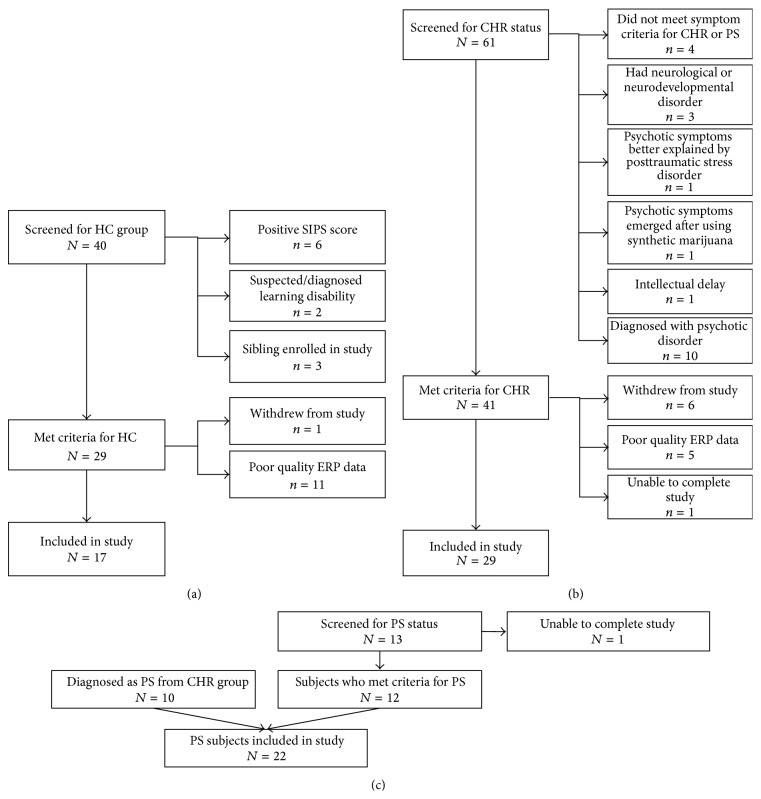
Flow charts depicting participants screened, reasons for exclusion, and number of participants retained for each clinical group. (a): healthy controls (HCs); (b): clinical high risk (CHR); (c): psychosis (PS).

Patients with PS (*n* = 22), patients with CHR (*n* = 29), and healthy controls (HCs; *n* = 17) between 5 and 17 years of age were recruited for this study. PS and CHR participants were drawn from three sources in the Boston area: (1) the psychiatry service at Boston Children's Hospital; (2) the Commonwealth Research Center (CRC, PI L. J. Seidman); and (3) the Social Neuroscience and Psychopathology Laboratory (SNAP Lab, PI C. Hooker) at Harvard University. The Structured Interview for Psychosis-risk Syndromes (SIPS; described in Section 2.2.1(2)) was used to determine whether PS or CHR syndrome criteria were met [49]. For each potential PS participant, the Schedule for Affective Disorders and Schizophrenia for School-Age Children-Present and Lifetime Version (K-SADS-PL) [50] (described in Section 2.2.1(1)) was utilized along with clinical reports from the participant's treating psychiatrist to determine a specific diagnosis using DSM-IV criteria. The 22 PS participants met criteria for the following psychotic diagnoses: schizophrenia (*n* = 7), schizoaffective disorder (*n* = 8), schizophreniform disorder (*n* = 3), bipolar disorder with psychotic features (*n* = 2), and major depression with psychotic features (*n* = 2). HCs were identified through advertisements and word of mouth. To qualify as HCs for the study, participants could not meet CHR criteria or have a current or past Axis I diagnosis. They also could not have any first-, second-, or third-degree biological relative with a psychotic disorder. Exclusion criteria for all participants included a lifetime diagnosis of substance abuse or dependence, neurological disease (e.g., epilepsy) or head injury, medical illness with cognitive sequelae, sensory impairments, or intellectual disability.  Figure 1 displays the results of the screening process (described in Section 2.2.1).

### 2.2. Procedures and Measures

A screening assessment was administered to confirm study eligibility and determine clinical group assignment. Eligible participants were then invited to complete demographic and clinical interviews/questionnaires and an auditory ERP paradigm. Boston Children's Hospital's Institutional Review Board approved all procedures. Participants provided assent, and a parent or legal guardian provided written informed consent.

#### 2.2.1. Screening Assessment

To determine study eligibility and group status, participants were administered a screening assessment, which consisted of the K-SADS-PL [50], the SIPS [49], and the Scale of Prodromal Symptoms (SOPS) [49]. If the ERP paradigm visit occurred more than one month after the screening assessment, the SIPS/SOPS were readministered to confirm clinical group assignment (i.e., to determine if any CHR participants had progressed to psychosis and to ensure that no HC had developed CHR or PS symptoms). No participant was reclassified based upon reassessment.

*(1) Schedule for Affective Disorders and Schizophrenia for School-Age Children-Present and Lifetime Version (K-SADS-PL) [50].* The K-SADS-PL is a reliable and validated semistructured interview widely used to diagnose mood disorders, anxiety disorders, substance abuse disorders, and psychotic disorders in individuals under the age of 18 years [50]. Participants and their parents/guardians were individually administered the K-SADS-PL by trained raters under the supervision of a board certified child and adolescent psychiatrist (JGH). Following standard procedures, children were asked to rate their symptoms, and parents/guardians were asked to rate their child's symptoms. For each participant, final diagnostic ratings were derived that considered both the child and parent/guardian scores [50].

*(2) Structured Interview for Psychosis-Risk Syndromes (SIPS) and the Scale of Prodromal Symptoms (SOPS) [49]*. The SIPS is an assessment instrument developed to operationally define psychosis disorder diagnoses, and the SOPS qualitatively rates symptom severity for positive and negative symptoms for patients prodromal for psychosis. Both measures have established predictive validity and excellent interrater reliability. Participants were administered the SIPS and the SOPS. Ratings were used to assess current or past psychosis and CHR status and to rate positive and negative symptom severity. SIPS/SOPS raters were trained and certified by Yale University's PRIME Research Clinic, and several attended North American Prodromal Longitudinal Study (NAPLS-2) SIPS interview reviews for 9 months. Sixty-six participants were administered the SIPS/SOPS by study staff; SIPS/SOPS scores for two participants were provided by their referral source (CRC or SNAP Lab), as they had been obtained within 30 days of the ERP paradigm visit.

#### 2.2.2. Demographic and Clinical Assessment

Race, ethnicity, date of birth, medical and psychiatric history, medication usage, and school functioning were determined from parent/guardian interview and record review. Intellectual disability was ruled out if (a) previous IQ testing results were >70 for full scale or verbal or performance IQ, (b) school functioning was at grade level without special education services, or (c) study staff administration of the Scales of Independent Behavior-Revised (SIB-R) [51], a comprehensive, norm-referenced assessment of functional level, indicated normal functioning.

#### 2.2.3. Auditory ERP Paradigm

Following a 10-minute baseline, EEG recordings were collected with an EGI 128-channel Geodesic Net System (Electrical Geodesics Inc., Eugene, OR) while the participant was seated in a quiet, electrically shielded room with eyes closed to reduce eye-movement artifact. Auditory stimuli were presented with TDH-49P headphones. To facilitate state stabilization, all participants viewed an age-appropriate video with the sound muted during presentation of the auditory stimuli.

The auditory stimuli were 450 identical sinusoidal tones of 1000 Hz constructed digitally using a sine wave function at 44,000 samples per second. Each tone was 50 ms long with a 0.005-second onset and offset ramp. After digital-to-analog conversion, the waveforms were reduced to within audible range (70 dB SPL) and routed to ear inserts, played binaurally with a randomly determined variable 1800–2600 ms interstimulus interval (Noesis software) to avoid rhythm artifact.

Trained staff visually edited data for movement and electrode artifact, eyeblink storms, state changes, and muscle activity. Automatic eyeblink and eye movement artifact removal procedures were then implemented using BESA Research 6.0 software (BESA GmbH, Gräfelfing, Germany). For each participant, the average N100 amplitude to the first 150 sinusoidal tones and to the last 150 sinusoidal tones was calculated. The N100 response was measured at the left frontal EEG position Fc1, which incorporates the left temporal lobe dipole (between Fc1 and Tp9 electrode positions) to provide the maximum N100 signal [52]. For each tone, only data with frequencies between 0.53 Hz and 50 Hz that fell within the averaging epoch (−500 ms to 500 ms) and passed BESA's amplitude filter (set from 150 *μ*V to 250 *μ*V) were used. To calculate the plasticity of participants' N100 generating mechanism, the mean amplitude of each participant's N100 during the last 150 tones was subtracted from the mean amplitude of the N100 during the first 150 tones, providing a measure of the extent of N100 attenuation.

### 2.3. Data Analysis Plan

Differences among clinical groups on demographic and clinical characteristics were tested using the Freeman-Halton extension of Fisher's exact tests for categorical variables and ANOVAs for continuous variables. Significant differences were followed by 2 × 2 Fisher's exact tests with Bonferroni corrected *p* threshold for categorical variables and Student-Newman-Keuls (SNK) tests for continuous variables.

Linear regression models were used, consistent with methods of similar analyses [33], to examine the effect of clinical group status on the plasticity measure (i.e., the mean amplitude of the N100 during the last 150 tones subtracted from the mean amplitude of the N100 during the first 150 tones) in the full sample and then separately in the younger (5 to 12 years old) and older (13 to 17 years old) subgroups. Prior to conducting these analyses, potential covariates, including age, gender, handedness, first-degree family history of mental illness (psychosis, nonpsychotic major depression, and nonpsychotic bipolar disorder), and medication usage (antipsychotics, antidepressants, mood stabilizers, benzodiazepines, and stimulants), were individually regressed against the plasticity measure in the full sample. Each variable that reached a significance level of *p* < .10 in its individual regression was included in the linear regression model testing the effect of clinical group status on plasticity. Variables were then removed using backward elimination with a threshold of *p* ≥ .10 to produce the final linear regression model. In the subsequent models stratified by participant age, only covariates that achieved significance (*p* < .05) in the final linear regression model on the full sample were included to minimize the likelihood of chance spurious results.

Following testing of the effects of clinical group on the plasticity measure, linear regression analyses were run that added the mean initial N100 amplitude (i.e., the mean N100 amplitude during the first 150 tones) as a covariate, as research in adults suggests that the N100 response is blunted in CHR and SZ [31–36]. This process minimized the risk of capitalizing on floor effects by testing whether any clinical group differences in plasticity were driven by initial N100 blunting in the CHR and/or PS groups, reducing the potential for attenuation over repeated presentations of the stimulus.

For all linear regression models in which clinical group emerged as a significant predictor, we confirmed the appropriateness of using a linear model by running ANOVAs to test deviation from linearity and by examining the residuals to ensure homoscedasticity and normal distributions (results not presented). Follow-up pairwise comparisons specified clinical group differences. For all analyses, *p* < .05 was considered statistically significant except where Bonferroni correction was used, as indicated below.

## 3. Results

### 3.1. Descriptive Data

Table 1 depicts the distributions of study variables across clinical groups. ANOVAs revealed significant differences between groups on age [*F*(2,65) = 4.493, *p* < .015] and SIB-R scaled scores [*F*(2,54) = 8.366, *p* = .001]. Fisher's exact tests revealed group differences on gender (*p* = .007) and usage of antipsychotics (*p* < .001) and antidepressants (*p* = .003). Table 1 specifies the significant pairwise group differences. Clinical groups did not differ on the remaining variables (*p*s ≥ .12).

### 3.2. Plasticity by Clinical Group

**Figure 2:**
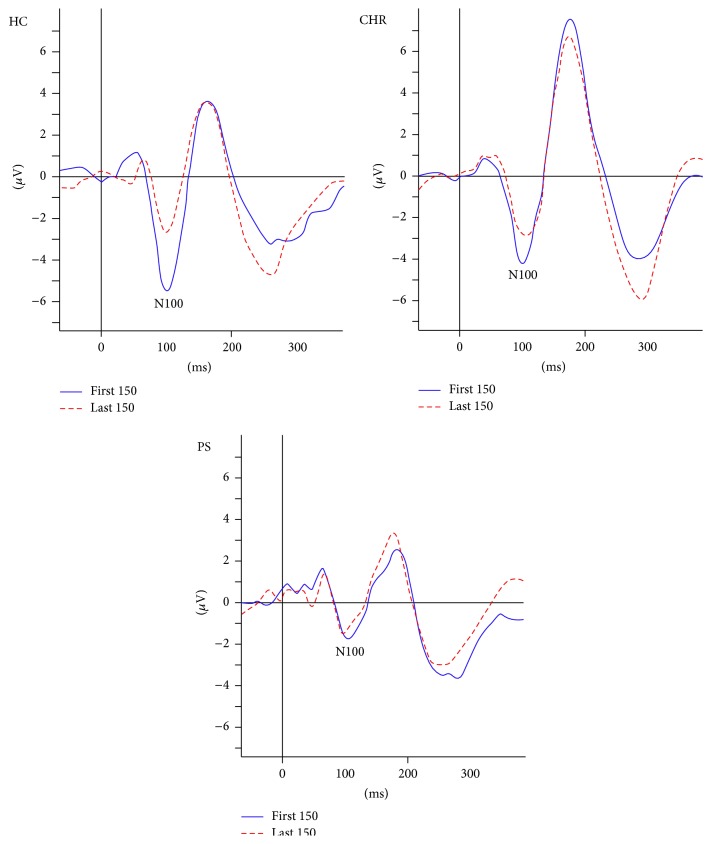
Average auditory N100 response for first and last 150 tones for full sample by clinical group. Auditory stimulus administered at 0 milliseconds. HC: healthy control; CHR: clinical high risk; PS: psychosis. HC > CHR, PS on the difference in the average N100 response to first versus last 150 tones.

In linear regression analyses in which potential covariates were individually regressed on the plasticity measure, antipsychotic usage and first-degree family history of nonpsychotic major depression met the *p* < .10 threshold and were therefore included in the linear regression analyses testing the effect of clincial group status on plasticity. In a linear regression analysis including clinical group status, antipsychotic usage, and first-degree family history of nonpsychotic major depression, backward elimination procedures resulted in antipsychotic usage dropping out; the overall model was significant, *R* ^2^ = .22, *F*(2,58) = 7.93, and *p* = .001, with a significant effect for clinical group status, *β* = −0.38, *t* = −3.22, and *p* = .002, and a marginal effect for family history of nonpsychotic major depression, *β* = −0.22, *t* = −1.82, and *p* = .073. From the first 150 tones to the last 150 tones, the N100 decreased by a mean of 2.32 *μ*V (2.45) among the HC group, 0.69 *μ*V (1.17) among the CHR group, and 0.42 *μ*V (1.60) among the PS group (Figure 2). Follow-up pairwise analyses revealed that clinical group status was a significant predictor of plasticity when comparing the HC and CHR groups, *β* = −0.46, *t* = −3.42, and *p* = .001, and the HC and PS groups, *β* = −0.47, *t* = −3.23, and *p* = .003, but not when comparing the CHR and PS groups, *β* = −0.10, *t* = −0.69, and *p* = .491.

Secondary analyses added the initial N100 amplitude (i.e., the mean N100 amplitude for the first 150 tones) to determine if initial blunting of the N100 response accounted for clinical group differences in plasticity. When initial N100 amplitude, clinical group status, antipsychotic usage, and family history of nonpsychotic major depression were included in the linear regression model, only initial N100 amplitude survived the backward eliminination procedures, *β* = 0.60, *t* = 5.76, and *p* < .001. Initial N100 amplitude explained a large percentage of the variance in plasticity, *R* ^2^ = .32, *F*(1,66) = 30.98, and *p* < .001. Follow-up pairwise analyses revealed that, when comparing the HC and CHR groups, both initial N100 amplitude, *β* = 0.49, *t* = 4.13, and *p* < .001, and clinical group status, *β* = −0.33, *t* = −2.80, and *p* = .008, were significant in predicting plasticity, *R* ^2^ = .43, *F*(2,43) = 16.48, and *p* < .001. When comparing the HC and PS groups, initial N100 amplitude, *β* = 0.58, *t* = 4.07, and *p* < .001, was significant in predicting plasticity, but clinical group status was not, *β* = −0.17, *t* = −1.22, and *p* = .231; *R* ^2^ = .44, *F*(2,36) = 15.67, and *p* < .001. Similarly, when comparing the CHR and PS groups, initial N100 amplitude, *β* = 0.38, *t* = 2.58, and *p* = .013, was significant in predicting plasticity, but clinical group status was not, *β* = 0.64, *t* = 0.43, and *p* = .667; *R* ^2^ = .13, *F*(2,48) = 3.60, and *p* = .035.

### 3.3. Plasticity by Clinical Group and Age

The interaction between age (as a continuous measure) and clinical group status was not significant in predicting the plasticity measure, *p* = .583.

#### 3.3.1. Plasticity by Clinical Group among Younger Participants (5 to 12 Years)

In analyses conducted separately for participants between 5 and 12 years of age, there was a significant decrease in the plasticity measure across clinicial groups: From the first 150 tones to the last 150 tones, the N100 decreased by a mean of 2.16 *μ*V (0.68) among the HC group (*n* = 10), 0.58 *μ*V (1.30) among the CHR group (*n* = 9), and 0.15 *μ*V (1.65) among the PS group (*n* = 15), *R* ^2^ = .20, *F*(1,32) = 7.98, and *p* = .008. Follow-up pairwise analyses revealed that clinical group status approached signficance as a predictor of plasticity when comparing the HC and CHR groups, *β* = −0.43, *t* = −1.95, and *p* = .068, and was a significant predictor when comparing the HC and PS groups, *β* = −0.49, *t* = −2.69, and *p* = .013. Clinicial group status was not a significant predictor when comparing the CHR and PS groups, *β* = −0.14, *t* = −0.67, and *p* = .513.

When initial N100 amplitude was added to the model predicting plasticity, it was significant, *β* = 0.35, *t* = 2.06, and *p* = .048, and clinical group status approached significance, *β* = −0.29, *t* = −1.72, and *p* = .095. In follow-up pairwise analyses comparing the HC and CHR groups, initial N100 amplitude was significant, *β* = 0.45, *t* = 2.26, and *p* = .038, and clinical group status approached significance, *β* = −0.36, *t* = −1.83, and *p* = .085. When comparing the HC and PS groups, initial N100 amplitude approached significance, *β* = 0.39, *t* = 1.94, and *p* = .065, and clinical group status was not significant, *β* = −0.29, *t* = −1.47, and *p* = .156; when clinical group status was removed, initial N100 amplitude emerged as a significant predictor, *β* = 0.54, *t* = 3.05, and *p* = .006. When comparing the CHR and PS groups, neither clinical group, *β* = −0.03, *t* = −0.15, and *p* = .885, nor initial N100 amplitude, *β* = 0.28, *t* = 1.22, and *p* = .237, was predictive of plasticity.

#### 3.3.2. Plasticity by Clinical Group among Older Participants (13 to 17 Years)

Among participants (13 to 17 years of age), the plasticity measure was greatest in the HC group but did not decrease in a linear fashion across clinicial groups: From the first 150 tones to the last 150 tones, the N100 decreased by a mean of 2.49 *μ*V (2.15) among the HC group (*n* = 7), 0.73 *μ*V (1.13) among the CHR group (*n* = 20), and 1.00 *μ*V (1.40) among the PS group (*n* = 7), *R* ^2^ = .10, *F*(1,32) = 3.47, and *p* = .072. Therefore, follow-up analyses were not conducted for the older subgroup.

## 4. Discussion

The goal of this study was to examine changes in the N100 response to repeated auditory stimulation as a potential biomarker sensitive to the neurological dysfunction that underlies clinical high risk (CHR) and progression to psychosis (PS). To the best of our knowledge, this is the first study to examine changes in the N100 response among a pediatric sample of patients at risk for or with PS. The findings suggest that impaired plasticity in the N100 is evident among both CHR and PS patients, particularly among younger (i.e., aged 5 to 12 years) patients, and is largely determined by decreased initial N100 amplitude in these patient groups. When analyses were conducted using the full sample, results showed a decrease in plasticity across clinical groups, with the HC group showing the greatest change in the N100 response across trials, followed by the CHR group, and then the PS group. Follow-up analyses showed significant differences in plasticity between the HC and CHR groups and the HC and PS groups, though these differences were diminished to nonsignificance for the latter comparison when the initial N100 response was considered. Only the initial N100 response accounted for differences between the CHR and PS groups.

When considered separately, the younger subgroup (5 to 12 years) demonstrated a decrease in plasticity across clinical groups from HC to CHR to PS. In follow-up analyses, the difference in the plasticity measure was significant between the HC and PS groups and approached significance between the HC and CHR groups. When initial N100 amplitude was considered, the plasticity difference between the HC and PS groups was partially explained by the decreased initial N100 amplitude of the PS patients. In the older subgroup, plasticity did not decrease in a linear fashion across clinical groups. Overall, the findings showed greater attenuation in the N100 repetition suppression response among the CHR and PS groups compared to the HC group, particularly among the younger subgroup. Notably, the CHR and PS groups did not differ in any of the pairwise analyses, suggesting that plasticity deficits associated with psychosis that are detectable via this measure are present early, during the CHR stage.

The findings also suggest that plasticity impairments among CHR and PS participants were largely driven by a decreased initial N100 response. Evidence for a blunted N100 response is consistent with studies in adults [4, 28, 31–36]. Possible explanations for this pattern of results include that the plasticity mechanisms measured by N100 repetition suppression were saturated before the repetition of the auditory stimulus, that these plasticity mechanisms are defective, whether due to NMDAR receptor mediated dysfunction or some other mechanism, that there is decreased sensory responsiveness that leads to underactivation of the plasticity mechanisms, and/or that there is a decreased number of synapses available to be altered by repetition of the sensory response, that is, a floor effect. The final hypothesis is consistent with the observation of low spine densities on the basilar dendrites of pyramidal neurons and decreased neuropil in postmortem cortical samples from patients with SZ, especially in cortical layers subserving corticocortical and thalamocortical connectivity [53]. This hypothesis is also consistent with findings in animal models of SZ (e.g., the NLHV rat model) that have demonstrated reduced numbers of neurons in the auditory cortex [41].

This study has limitations. Its small sample size restricted statistical power, particularly for subgroup analyses. Several of the tests for pairwise clinical group differences approached significance and may have achieved significance in a larger sample. The sample was almost exclusively non-Hispanic White, and the PS subsample was largely male. We attempted to minimize the role of intelligence in contributing to group differences on N100 measures by excluding individuals with intellectual disability. However, future studies should match participants on IQ and/or control for IQ in analyses, as intellectual capabilities are associated with N100 responses [54]. The CHR group was older than the HC and PS groups; however, age was not a significant predictor of the plasticity measure. Although we considered medication usage as a covariate in analyses, we cannot rule out medication effects given that a significant proportion of the PS and CHR patients were taking medication and no HC participants were medicated. Another limitation relates to the assessment of CHR in pediatric populations. Although childhood onset of prodromal symptoms is not rare [55], identification of CHR in pediatric populations is less reliable than in adults [56]. Furthermore, the predictive validity of the SIPS, particularly in children under the age of 10 years, is not established. Finally, the PS group was not limited to DSM-IV SZ due to (a) difficulty in determining if patients with early psychosis would settle into a categorical diagnosis of SZ or an affective psychosis [57, 58] and (b) increasing biological evidence for heterogeneity among patients with SZ and for pleiotropy in the phenotypic expressions of SZ risk alleles [59]. Consequently, we employed a Research Domain Criteria (RDoC) approach to participant selection, as recently advocated by the National Institute of Mental Health [60].

## 5. Conclusions

The current findings offer evidence of deficits in repetition suppression of the N100 response in early onset (<18 years old) and very early onset (<13 years old) CHR and PS. These findings warrant further study to determine the usefulness of repetition suppression of the N100 response as a biomarker of deficits in brain plasticity in CHR and PS. Furthermore, these cross-sectional findings indicate the need to follow participants longitudinally to assess the stability of the N100 plasticity measure in CHR and PS patients and to determine if more pronounced sensory adaptation deficits predict which CHR patients progress to psychosis. Additional study is also needed to determine whether this measure is responsive to target engagement by therapies meant to reverse plasticity deficits that may underlie the development of psychosis. If supported by longitudinal studies, measurements of sensory adaptation deficits may be useful outcomes for early clinical trials of proposed therapies for CHR and PS.

## Figures and Tables

**Table 1:** Demographic and clinical characteristics of clinical groups.

Variable	Clinical group								
	PS			CHR			HC		
	(*n* = 22)			(*n* = 29)			(*n* = 17)		
	%	M	SD	%	M	SD	%	M	SD
Demographics									
Male (% male)	86.4^a^			44.8^b^			52.9		
Age (years)		11.4^a^	2.8		13.5^b^	2.7		11.0^a^	4.2
Race/ethnicity (% non-Hispanic White)	95.2			79.3			94.1		
Handedness (% left-handed)	13.6			6.9			5.9		
SIB-R SS^c^		75.6^a^	27.9		89.6^a^	13.0		107.1^b^	20.9
First-degree family mental health history^d^									
Psychosis	14.3			4.4			0		
Nonpsychotic major depression	14.3			21.7			0		
Nonpsychotic bipolar disorder	4.8			17.4			11.8		
Medication use at assessment									
Antipsychotic(s)	50.0^a^			27.6			0^b^		
Antidepressant(s)	45.5^a^			31.1			0^b^		
Mood stabilizer(s)	18.2			6.9			0		
Benzodiazepine(s)	4.5			3.5			0		
Stimulant(s)	4.5			0			0		

Note: PS, psychosis; CHR, clinical high risk; HC, healthy control; SIB-R SS, Scales of Independent Behavior-Revised scaled scores.

^a,b^Groups noted by different superscripted letters were significantly different in post hoc pairwise Student-Newman-Keuls (SNK) tests for continuous variables and Bonferroni corrected (*p* < .017) Fisher's exact tests for categorical variables. ^c^The SIB-R was administered to 20 PS participants, 21 CHR participants, and 16 HC participants. ^d^One PS participant and six CHR participants were unable to provide information about family history of mental illness because they had limited contact with their biological parents or their adoptive parents were unsure of mental illness history among first-degree biological relatives.
